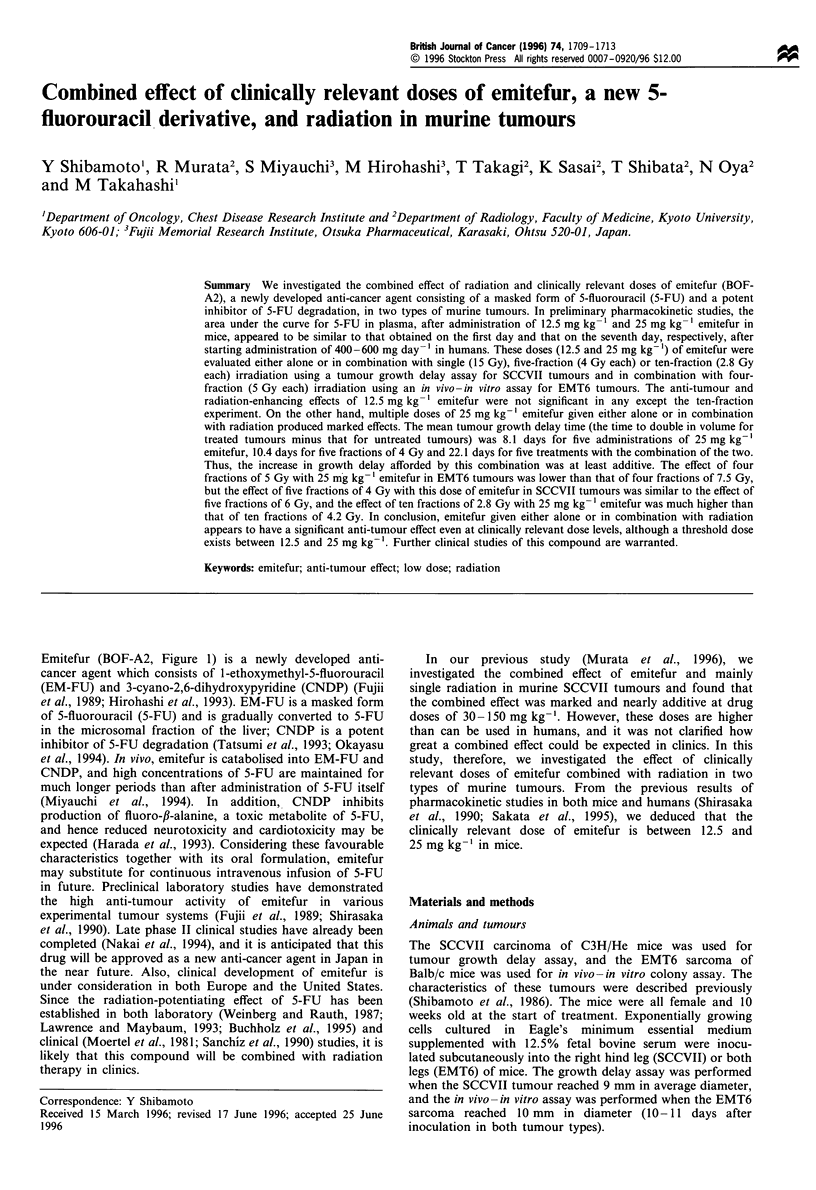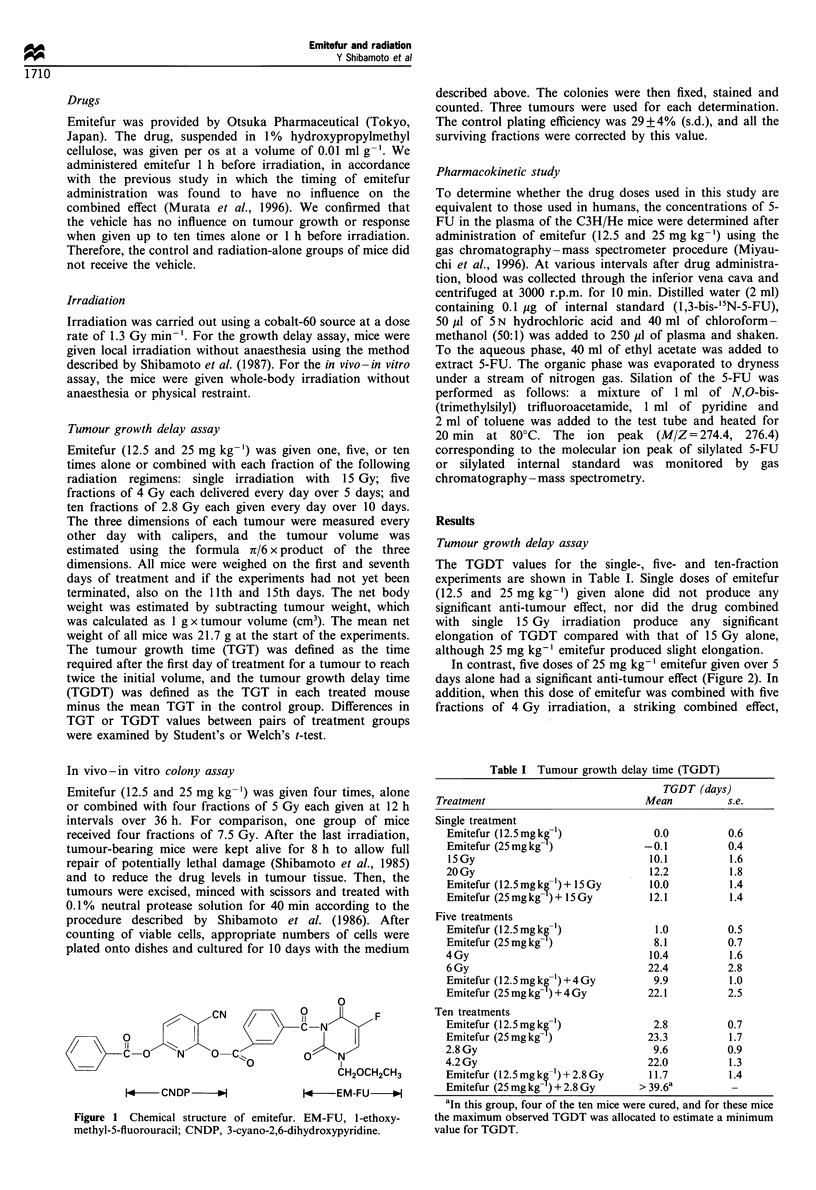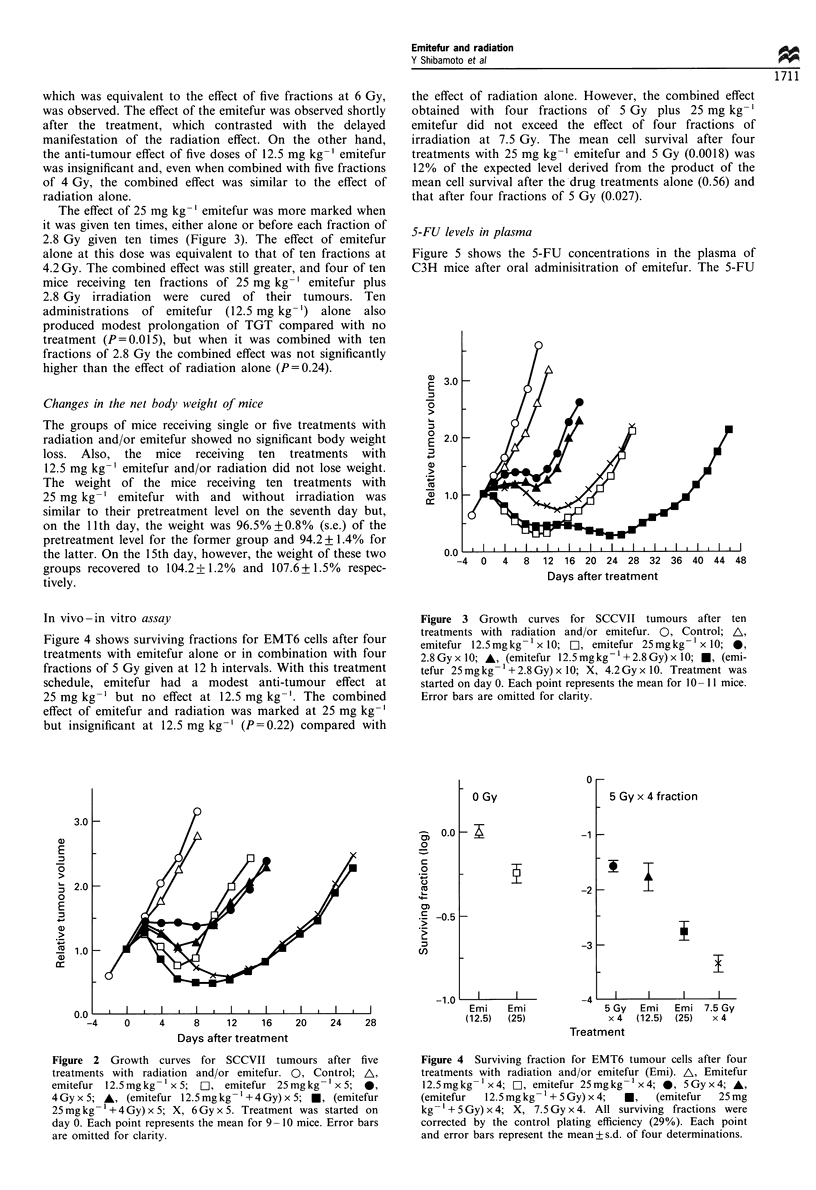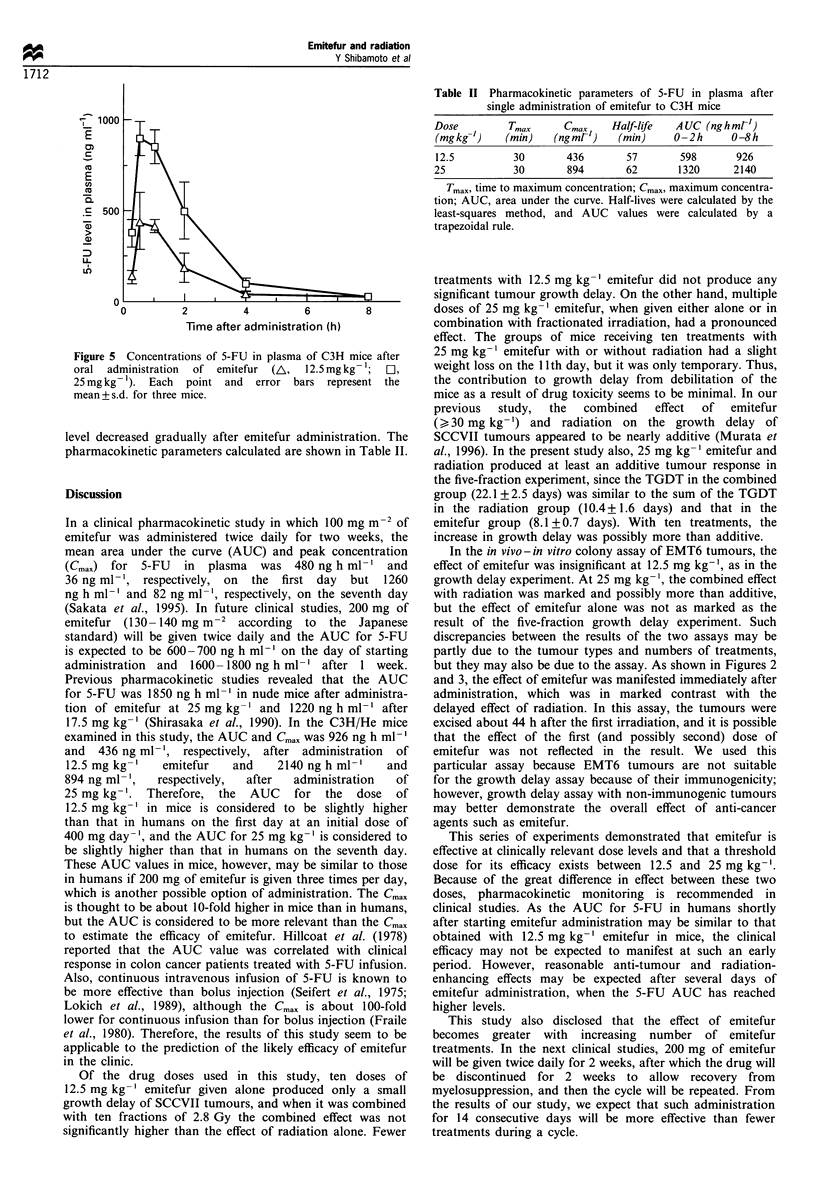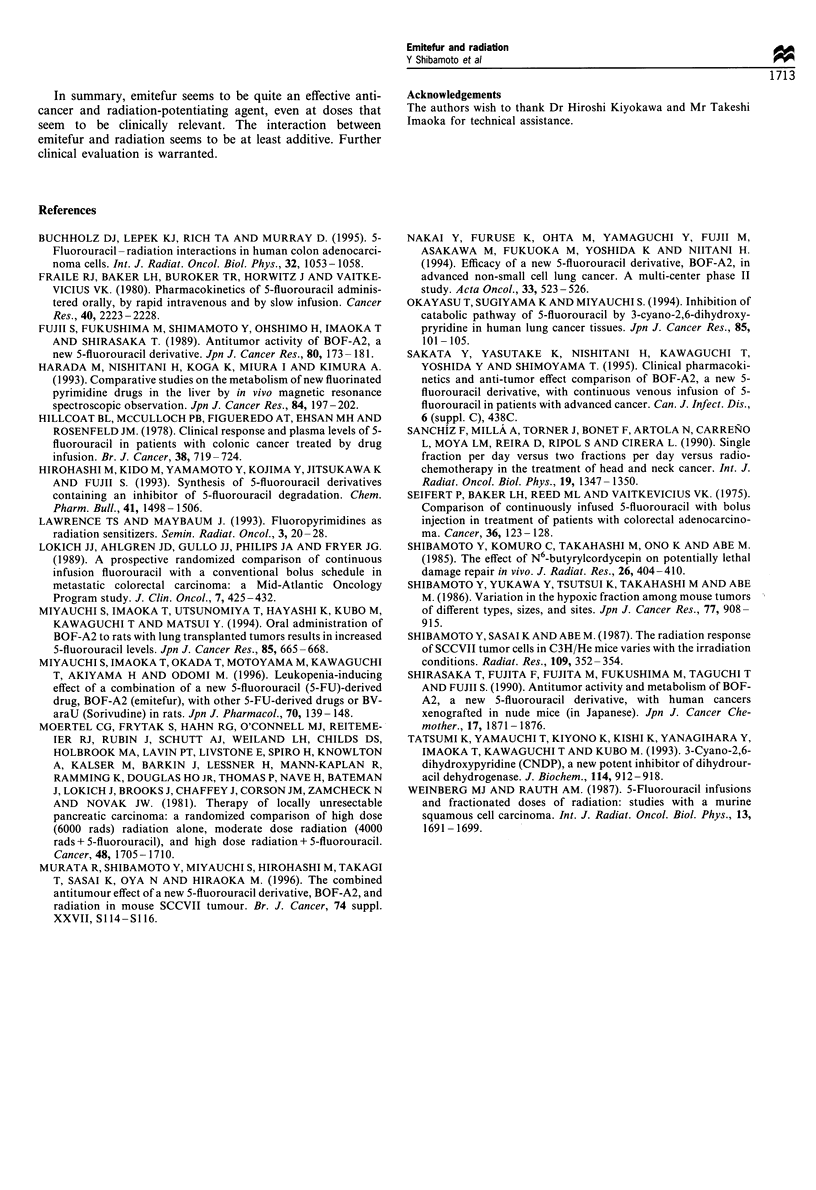# Combined effect of clinically relevant doses of emitefur, a new 5-fluorouracil derivative, and radiation in murine tumours.

**DOI:** 10.1038/bjc.1996.619

**Published:** 1996-12

**Authors:** Y. Shibamoto, R. Murata, S. Miyauchi, M. Hirohashi, T. Takagi, K. Sasai, T. Shibata, N. Oya, M. Takahashi

**Affiliations:** Department of Oncology, Faculty of Medicine, Kyoto University, Japan.

## Abstract

We investigated the combined effect of radiation and clinically relevant doses of emitefur (BOF-A2), a newly developed anti-cancer agent consisting of a masked form of 5-fluorouracil (5-FU) and a potent inhibitor of 5-FU degradation, in two types of murine tumours. In preliminary pharmacokinetic studies, the area under the curve for 5-FU in plasma, after administration of 12.5 mg kg-1 and 25 mg kg-1 emitefur in mice, appeared to be similar to that obtained on the first day and that on the seventh day, respectively, after starting administration of 400-600 mg day-1 in humans. These doses (12.5 and 25 mg kg-1) of emitefur were evaluated either alone or in combination with single (15 Gy), five-fraction (4 Gy each) or ten-fraction (2.8 Gy each) irradiation using a tumour growth delay assay for SCCVII tumours and in combination with four-fraction (5 Gy each) irradiation using an in vivo-in vitro assay for EMT6 tumours. The anti-tumour and radiation-enhancing effects of 12.5 mg kg-1 emitefur were not significant in any except the ten-fraction experiment. On the other hand, multiple doses of 25 mg kg-1 emitefur given either alone or in combination with radiation produced marked effects. The mean tumour growth delay time (the time to double in volume for treated tumours minus that for untreated tumours) was 8.1 days for five administrations of 25 mg kg-1 emitefur. 10.4 days for five fractions of 4 Gy and 22.1 days for five treatments with the combination of the two. Thus, the increase in growth delay afforded by this combination was at least additive. The effect of four fractions of 5 Gy with 25 mg kg-1 emitefur in EMT6 tumours was lower than that of four fractions of 7.5 Gy, but the effect of five fractions of 4 Gy with this dose of emitefur in SCCVII tumours was similar to the effect of five fractions of 6 Gy, and the effect of ten fractions of 2.8 Gy with 25 mg kg-1 emitefur was much higher than that of ten fractions of 4.2 Gy. In conclusion, emitefur given either alone or in combination with radiation appears to have a significant anti-tumour effect even at clinically relevant dose levels, although a threshold dose exists between 12.5 and 25 mg kg-1. Further clinical studies of this compound are warranted.


British Journal of Cancer (1996) 74, 1709-1713
© 1996 Stockton Press All rights reserved 0007-0920/96 $12.00

### Combined effect of clinically relevant doses of emitefur, a new 5-fluorouracil derivative, and radiation in murine tumours

Y Shibamoto[1], R Murata[2], S Miyauchi[3], M Hirohashi[3], T Takagi[2], K Sasai[2], T Shibata[2], N Oya[2] and M Takahashi[1]

[1]*Department of Oncology, Chest Disease Research Institute and* [2]*Department of Radiology, Faculty of Medicine, Kyoto University, Kyoto 606-01;* [3]*Fujii Memorial Research Institute, Otsuka Pharmaceutical, Karasaki, Ohtsu 520-01, Japan.*

**Summary** We investigated the combined effect of radiation and clinically relevant doses of emitefur (BOF-A2), a newly developed anti-cancer agent consisting of a masked form of 5-fluorouracil (5-FU) and a potent inhibitor of 5-FU degradation, in two types of murine tumours. In preliminary pharmacokinetic studies, the area under the curve for 5-FU in plasma, after administration of 12.5 mg $kg^{-1}$ and 25 mg $kg^{-1}$ emitefur in mice, appeared to be similar to that obtained on the first day and that on the seventh day, respectively, after starting administration of 400-600 mg $day^{-1}$ in humans. These doses (12.5 and 25 mg $kg^{-1}$) of emitefur were evaluated either alone or in combination with single (15 Gy), five-fraction (4 Gy each) or ten-fraction (2.8 Gy each) irradiation using a tumour growth delay assay for SCCVII tumours and in combination with four-fraction (5 Gy each) irradiation using an *in vivo-in vitro* assay for EMT6 tumours. The anti-tumour and radiation-enhancing effects of 12.5 mg $kg^{-1}$ emitefur were not significant in any except the ten-fraction experiment. On the other hand, multiple doses of 25 mg $kg^{-1}$ emitefur given either alone or in combination with radiation produced marked effects. The mean tumour growth delay time (the time to double in volume for treated tumours minus that for untreated tumours) was 8.1 days for five administrations of 25 mg $kg^{-1}$ emitefur, 10.4 days for five fractions of 4 Gy and 22.1 days for five treatments with the combination of the two. Thus, the increase in growth delay afforded by this combination was at least additive. The effect of four fractions of 5 Gy with 25 mg $kg^{-1}$ emitefur in EMT6 tumours was lower than that of four fractions of 7.5 Gy, but the effect of five fractions of 4 Gy with this dose of emitefur in SCCVII tumours was similar to the effect of five fractions of 6 Gy, and the effect of ten fractions of 2.8 Gy with 25 mg $kg^{-1}$ emitefur was much higher than that of ten fractions of 4.2 Gy. In conclusion, emitefur given either alone or in combination with radiation appears to have a significant anti-tumour effect even at clinically relevant dose levels, although a threshold dose exists between 12.5 and 25 mg $kg^{-1}$. Further clinical studies of this compound are warranted.

**Keywords:** emitefur; anti-tumour effect; low dose; radiation

Emitefur (BOF-A2, Figure 1) is a newly developed anti-cancer agent which consists of 1-ethoxymethyl-5-fluorouracil (EM-FU) and 3-cyano-2,6-dihydroxypyridine (CNDP) (Fujii *et al.*, 1989; Hirohashi *et al.*, 1993). EM-FU is a masked form of 5-fluorouracil (5-FU) and is gradually converted to 5-FU in the microsomal fraction of the liver; CNDP is a potent inhibitor of 5-FU degradation (Tatsumi *et al.*, 1993; Okayasu *et al.*, 1994). *In vivo*, emitefur is catabolised into EM-FU and CNDP, and high concentrations of 5-FU are maintained for much longer periods than after administration of 5-FU itself (Miyauchi *et al.*, 1994). In addition, CNDP inhibits production of fluoro-$\beta$-alanine, a toxic metabolite of 5-FU, and hence reduced neurotoxicity and cardiotoxicity may be expected (Harada *et al.*, 1993). Considering these favourable characteristics together with its oral formulation, emitefur may substitute for continuous intravenous infusion of 5-FU in future. Preclinical laboratory studies have demonstrated the high anti-tumour activity of emitefur in various experimental tumour systems (Fujii *et al.*, 1989; Shirasaka *et al.*, 1990). Late phase II clinical studies have already been completed (Nakai *et al.*, 1994), and it is anticipated that this drug will be approved as a new anti-cancer agent in Japan in the near future. Also, clinical development of emitefur is under consideration in both Europe and the United States. Since the radiation-potentiating effect of 5-FU has been established in both laboratory (Weinberg and Rauth, 1987; Lawrence and Maybaum, 1993; Buchholz *et al.*, 1995) and clinical (Moertel *et al.*, 1981; Sanchíz *et al.*, 1990) studies, it is likely that this compound will be combined with radiation therapy in clinics.

Correspondence: Y Shibamoto
Received 15 March 1996; revised 17 June 1996; accepted 25 June 1996

In our previous study (Murata *et al.*, 1996), we investigated the combined effect of emitefur and mainly single radiation in murine SCCVII tumours and found that the combined effect was marked and nearly additive at drug doses of 30-150 mg $kg^{-1}$. However, these doses are higher than can be used in humans, and it was not clarified how great a combined effect could be expected in clinics. In this study, therefore, we investigated the effect of clinically relevant doses of emitefur combined with radiation in two types of murine tumours. From the previous results of pharmacokinetic studies in both mice and humans (Shirasaka *et al.*, 1990; Sakata *et al.*, 1995), we deduced that the clinically relevant dose of emitefur is between 12.5 and 25 mg $kg^{-1}$ in mice.

### Materials and methods

### *Animals and tumours*

The SCCVII carcinoma of C3H/He mice was used for tumour growth delay assay, and the EMT6 sarcoma of Balb/c mice was used for *in vivo-in vitro* colony assay. The characteristics of these tumours were described previously (Shibamoto *et al.*, 1986). The mice were all female and 10 weeks old at the start of treatment. Exponentially growing cells cultured in Eagle's minimum essential medium supplemented with 12.5% fetal bovine serum were inoculated subcutaneously into the right hind leg (SCCVII) or both legs (EMT6) of mice. The growth delay assay was performed when the SCCVII tumour reached 9 mm in average diameter, and the *in vivo-in vitro* assay was performed when the EMT6 sarcoma reached 10 mm in diameter (10-11 days after inoculation in both tumour types).

*Drugs*

Emitefur was provided by Otsuka Pharmaceutical (Tokyo, Japan). The drug, suspended in 1% hydroxypropylmethyl cellulose, was given per os at a volume of 0.01 ml $g^{-1}$. We administered emitefur 1 h before irradiation, in accordance with the previous study in which the timing of emitefur administration was found to have no influence on the combined effect (Murata *et al.*, 1996). We confirmed that the vehicle has no influence on tumour growth or response when given up to ten times alone or 1 h before irradiation. Therefore, the control and radiation-alone groups of mice did not receive the vehicle.

*Irradiation*

Irradiation was carried out using a cobalt-60 source at a dose rate of 1.3 Gy $min^{-1}$. For the growth delay assay, mice were given local irradiation without anaesthesia using the method described by Shibamoto *et al.* (1987). For the *in vivo–in vitro* assay, the mice were given whole-body irradiation without anaesthesia or physical restraint.

*Tumour growth delay assay*

Emitefur (12.5 and 25 mg $kg^{-1}$) was given one, five, or ten times alone or combined with each fraction of the following radiation regimens: single irradiation with 15 Gy; five fractions of 4 Gy each delivered every day over 5 days; and ten fractions of 2.8 Gy each given every day over 10 days. The three dimensions of each tumour were measured every other day with calipers, and the tumour volume was estimated using the formula $\pi/6 \times$ product of the three dimensions. All mice were weighed on the first and seventh days of treatment and if the experiments had not yet been terminated, also on the 11th and 15th days. The net body weight was estimated by subtracting tumour weight, which was calculated as 1 g $\times$ tumour volume ($cm^3$). The mean net weight of all mice was 21.7 g at the start of the experiments. The tumour growth time (TGT) was defined as the time required after the first day of treatment for a tumour to reach twice the initial volume, and the tumour growth delay time (TGDT) was defined as the TGT in each treated mouse minus the mean TGT in the control group. Differences in TGT or TGDT values between pairs of treatment groups were examined by Student's or Welch's *t*-test.

In vivo–in vitro *colony assay*

Emitefur (12.5 and 25 mg $kg^{-1}$) was given four times, alone or combined with four fractions of 5 Gy each given at 12 h intervals over 36 h. For comparison, one group of mice received four fractions of 7.5 Gy. After the last irradiation, tumour-bearing mice were kept alive for 8 h to allow full repair of potentially lethal damage (Shibamoto *et al.*, 1985) and to reduce the drug levels in tumour tissue. Then, the tumours were excised, minced with scissors and treated with 0.1% neutral protease solution for 40 min according to the procedure described by Shibamoto *et al.* (1986). After counting of viable cells, appropriate numbers of cells were plated onto dishes and cultured for 10 days with the medium described above. The colonies were then fixed, stained and counted. Three tumours were used for each determination. The control plating efficiency was 29±4% (s.d.), and all the surviving fractions were corrected by this value.

**Figure 1** Chemical structure of emitefur. EM-FU, 1-ethoxymethyl-5-fluorouracil; CNDP, 3-cyano-2,6-dihydroxypyridine.

*Pharmacokinetic study*

To determine whether the drug doses used in this study are equivalent to those used in humans, the concentrations of 5-FU in the plasma of the C3H/He mice were determined after administration of emitefur (12.5 and 25 mg $kg^{-1}$) using the gas chromatography–mass spectrometer procedure (Miyauchi *et al.*, 1996). At various intervals after drug administration, blood was collected through the inferior vena cava and centrifuged at 3000 r.p.m. for 10 min. Distilled water (2 ml) containing 0.1 $\mu$g of internal standard (1,3-bis-$^{15}$N-5-FU), 50 $\mu$l of 5 N hydrochloric acid and 40 ml of chloroform–methanol (50:1) was added to 250 $\mu$l of plasma and shaken. To the aqueous phase, 40 ml of ethyl acetate was added to extract 5-FU. The organic phase was evaporated to dryness under a stream of nitrogen gas. Silation of the 5-FU was performed as follows: a mixture of 1 ml of *N,O*-bis-(trimethylsilyl) trifluoroacetamide, 1 ml of pyridine and 2 ml of toluene was added to the test tube and heated for 20 min at 80°C. The ion peak ($M/Z$=274.4, 276.4) corresponding to the molecular ion peak of silylated 5-FU or silylated internal standard was monitored by gas chromatography–mass spectrometry.

### Results

*Tumour growth delay assay*

The TGDT values for the single-, five- and ten-fraction experiments are shown in Table I. Single doses of emitefur (12.5 and 25 mg $kg^{-1}$) given alone did not produce any significant anti-tumour effect, nor did the drug combined with single 15 Gy irradiation produce any significant elongation of TGDT compared with that of 15 Gy alone, although 25 mg $kg^{-1}$ emitefur produced slight elongation.

In contrast, five doses of 25 mg $kg^{-1}$ emitefur given over 5 days alone had a significant anti-tumour effect (Figure 2). In addition, when this dose of emitefur was combined with five fractions of 4 Gy irradiation, a striking combined effect,

**Table I** Tumour growth delay time (TGDT)

| Treatment | TGDT (days) Mean | s.e. |
|---|---|---|
| Single treatment | | |
| Emitefur (12.5 mg $kg^{-1}$) | 0.0 | 0.6 |
| Emitefur (25 mg $kg^{-1}$) | −0.1 | 0.4 |
| 15 Gy | 10.1 | 1.6 |
| 20 Gy | 12.2 | 1.8 |
| Emitefur (12.5 mg $kg^{-1}$) + 15 Gy | 10.0 | 1.4 |
| Emitefur (25 mg $kg^{-1}$) + 15 Gy | 12.1 | 1.4 |
| Five treatments | | |
| Emitefur (12.5 mg $kg^{-1}$) | 1.0 | 0.5 |
| Emitefur (25 mg $kg^{-1}$) | 8.1 | 0.7 |
| 4 Gy | 10.4 | 1.6 |
| 6 Gy | 22.4 | 2.8 |
| Emitefur (12.5 mg $kg^{-1}$) + 4 Gy | 9.9 | 1.0 |
| Emitefur (25 mg $kg^{-1}$) + 4 Gy | 22.1 | 2.5 |
| Ten treatments | | |
| Emitefur (12.5 mg $kg^{-1}$) | 2.8 | 0.7 |
| Emitefur (25 mg $kg^{-1}$) | 23.3 | 1.7 |
| 2.8 Gy | 9.6 | 0.9 |
| 4.2 Gy | 22.0 | 1.3 |
| Emitefur (12.5 mg $kg^{-1}$) + 2.8 Gy | 11.7 | 1.4 |
| Emitefur (25 mg $kg^{-1}$) + 2.8 Gy | >39.6[a] | – |

[a]In this group, four of the ten mice were cured, and for these mice the maximum observed TGDT was allocated to estimate a minimum value for TGDT.

which was equivalent to the effect of five fractions at 6 Gy, was observed. The effect of the emitefur was observed shortly after the treatment, which contrasted with the delayed manifestation of the radiation effect. On the other hand, the anti-tumour effect of five doses of 12.5 mg $kg^{-1}$ emitefur was insignificant and, even when combined with five fractions of 4 Gy, the combined effect was similar to the effect of radiation alone.

The effect of 25 mg $kg^{-1}$ emitefur was more marked when it was given ten times, either alone or before each fraction of 2.8 Gy given ten times (Figure 3). The effect of emitefur alone at this dose was equivalent to that of ten fractions at 4.2 Gy. The combined effect was still greater, and four of ten mice receiving ten fractions of 25 mg $kg^{-1}$ emitefur plus 2.8 Gy irradiation were cured of their tumours. Ten administrations of emitefur (12.5 mg $kg^{-1}$) alone also produced modest prolongation of TGT compared with no treatment ($P=0.015$), but when it was combined with ten fractions of 2.8 Gy the combined effect was not significantly higher than the effect of radiation alone ($P=0.24$).

### *Changes in the net body weight of mice*

The groups of mice receiving single or five treatments with radiation and/or emitefur showed no significant body weight loss. Also, the mice receiving ten treatments with 12.5 mg $kg^{-1}$ emitefur and/or radiation did not lose weight. The weight of the mice receiving ten treatments with 25 mg $kg^{-1}$ emitefur with and without irradiation was similar to their pretreatment level on the seventh day but, on the 11th day, the weight was 96.5% ± 0.8% (s.e.) of the pretreatment level for the former group and 94.2 ± 1.4% for the latter. On the 15th day, however, the weight of these two groups recovered to 104.2 ± 1.2% and 107.6 ± 1.5% respectively.

### In vivo–in vitro *assay*

Figure 4 shows surviving fractions for EMT6 cells after four treatments with emitefur alone or in combination with four fractions of 5 Gy given at 12 h intervals. With this treatment schedule, emitefur had a modest anti-tumour effect at 25 mg $kg^{-1}$ but no effect at 12.5 mg $kg^{-1}$. The combined effect of emitefur and radiation was marked at 25 mg $kg^{-1}$ but insignificant at 12.5 mg $kg^{-1}$ ($P=0.22$) compared with the effect of radiation alone. However, the combined effect obtained with four fractions of 5 Gy plus 25 mg $kg^{-1}$ emitefur did not exceed the effect of four fractions of irradiation at 7.5 Gy. The mean cell survival after four treatments with 25 mg $kg^{-1}$ emitefur and 5 Gy (0.0018) was 12% of the expected level derived from the product of the mean cell survival after the drug treatments alone (0.56) and that after four fractions of 5 Gy (0.027).

### *5-FU levels in plasma*

Figure 5 shows the 5-FU concentrations in the plasma of C3H mice after oral adminisitration of emitefur. The 5-FU

**Figure 3** Growth curves for SCCVII tumours after ten treatments with radiation and/or emitefur. ○, Control; △, emitefur 12.5 mg $kg^{-1}$ × 10; □, emitefur 25 mg $kg^{-1}$ × 10; ●, 2.8 Gy × 10; ▲, (emitefur 12.5 mg $kg^{-1}$ + 2.8 Gy) × 10; ■, (emitefur 25 mg $kg^{-1}$ + 2.8 Gy) × 10; X, 4.2 Gy × 10. Treatment was started on day 0. Each point represents the mean for 10–11 mice. Error bars are omitted for clarity.

**Figure 2** Growth curves for SCCVII tumours after five treatments with radiation and/or emitefur. ○, Control; △, emitefur 12.5 mg $kg^{-1}$ × 5; □, emitefur 25 mg $kg^{-1}$ × 5; ●, 4 Gy × 5; ▲, (emitefur 12.5 mg $kg^{-1}$ + 4 Gy) × 5; ■, (emitefur 25 mg $kg^{-1}$ + 4 Gy) × 5; X, 6 Gy × 5. Treatment was started on day 0. Each point represents the mean for 9–10 mice. Error bars are omitted for clarity.

**Figure 4** Surviving fraction for EMT6 tumour cells after four treatments with radiation and/or emitefur (Emi). △, Emitefur 12.5 mg $kg^{-1}$ × 4; □, emitefur 25 mg $kg^{-1}$ × 4; ●, 5 Gy × 4; ▲, (emitefur 12.5 mg $kg^{-1}$ + 5 Gy) × 4; ■, (emitefur 25 mg $kg^{-1}$ + 5 Gy) × 4; X, 7.5 Gy × 4. All surviving fractions were corrected by the control plating efficiency (29%). Each point and error bars represent the mean ± s.d. of four determinations.

**Figure 5** Concentrations of 5-FU in plasma of C3H mice after oral administration of emitefur (△, 12.5 mg kg$^{-1}$; □, 25 mg kg$^{-1}$). Each point and error bars represent the mean ± s.d. for three mice.

level decreased gradually after emitefur administration. The pharmacokinetic parameters calculated are shown in Table II.

### Discussion

In a clinical pharmacokinetic study in which 100 mg m$^{-2}$ of emitefur was administered twice daily for two weeks, the mean area under the curve (AUC) and peak concentration ($C_{max}$) for 5-FU in plasma was 480 ng h ml$^{-1}$ and 36 ng ml$^{-1}$, respectively, on the first day but 1260 ng h ml$^{-1}$ and 82 ng ml$^{-1}$, respectively, on the seventh day (Sakata *et al.*, 1995). In future clinical studies, 200 mg of emitefur (130–140 mg m$^{-2}$ according to the Japanese standard) will be given twice daily and the AUC for 5-FU is expected to be 600–700 ng h ml$^{-1}$ on the day of starting administration and 1600–1800 ng h ml$^{-1}$ after 1 week. Previous pharmacokinetic studies revealed that the AUC for 5-FU was 1850 ng h ml$^{-1}$ in nude mice after administration of emitefur at 25 mg kg$^{-1}$ and 1220 ng h ml$^{-1}$ after 17.5 mg kg$^{-1}$ (Shirasaka *et al.*, 1990). In the C3H/He mice examined in this study, the AUC and $C_{max}$ was 926 ng h ml$^{-1}$ and 436 ng ml$^{-1}$, respectively, after administration of 12.5 mg kg$^{-1}$ emitefur and 2140 ng h ml$^{-1}$ and 894 ng ml$^{-1}$, respectively, after administration of 25 mg kg$^{-1}$. Therefore, the AUC for the dose of 12.5 mg kg$^{-1}$ in mice is considered to be slightly higher than that in humans on the first day at an initial dose of 400 mg day$^{-1}$, and the AUC for 25 mg kg$^{-1}$ is considered to be slightly higher than that in humans on the seventh day. These AUC values in mice, however, may be similar to those in humans if 200 mg of emitefur is given three times per day, which is another possible option of administration. The $C_{max}$ is thought to be about 10-fold higher in mice than in humans, but the AUC is considered to be more relevant than the $C_{max}$ to estimate the efficacy of emitefur. Hillcoat *et al.* (1978) reported that the AUC value was correlated with clinical response in colon cancer patients treated with 5-FU infusion. Also, continuous intravenous infusion of 5-FU is known to be more effective than bolus injection (Seifert *et al.*, 1975; Lokich *et al.*, 1989), although the $C_{max}$ is about 100-fold lower for continuous infusion than for bolus injection (Fraile *et al.*, 1980). Therefore, the results of this study seem to be applicable to the prediction of the likely efficacy of emitefur in the clinic.

Of the drug doses used in this study, ten doses of 12.5 mg kg$^{-1}$ emitefur given alone produced only a small growth delay of SCCVII tumours, and when it was combined with ten fractions of 2.8 Gy the combined effect was not significantly higher than the effect of radiation alone. Fewer

**Table II** Pharmacokinetic parameters of 5-FU in plasma after single administration of emitefur to C3H mice

| *Dose (mg kg$^{-1}$)* | *$T_{max}$ (min)* | *$C_{max}$ (ng ml$^{-1}$)* | *Half-life (min)* | *AUC (ng h ml$^{-1}$) 0–2 h* | *0–8 h* |
|---|---|---|---|---|---|
| 12.5 | 30 | 436 | 57 | 598 | 926 |
| 25 | 30 | 894 | 62 | 1320 | 2140 |

$T_{max}$, time to maximum concentration; $C_{max}$, maximum concentration; AUC, area under the curve. Half-lives were calculated by the least-squares method, and AUC values were calculated by a trapezoidal rule.

treatments with 12.5 mg kg$^{-1}$ emitefur did not produce any significant tumour growth delay. On the other hand, multiple doses of 25 mg kg$^{-1}$ emitefur, when given either alone or in combination with fractionated irradiation, had a pronounced effect. The groups of mice receiving ten treatments with 25 mg kg$^{-1}$ emitefur with or without radiation had a slight weight loss on the 11th day, but it was only temporary. Thus, the contribution to growth delay from debilitation of the mice as a result of drug toxicity seems to be minimal. In our previous study, the combined effect of emitefur (⩾30 mg kg$^{-1}$) and radiation on the growth delay of SCCVII tumours appeared to be nearly additive (Murata *et al.*, 1996). In the present study also, 25 mg kg$^{-1}$ emitefur and radiation produced at least an additive tumour response in the five-fraction experiment, since the TGDT in the combined group (22.1 ± 2.5 days) was similar to the sum of the TGDT in the radiation group (10.4 ± 1.6 days) and that in the emitefur group (8.1 ± 0.7 days). With ten treatments, the increase in growth delay was possibly more than additive.

In the *in vivo–in vitro* colony assay of EMT6 tumours, the effect of emitefur was insignificant at 12.5 mg kg$^{-1}$, as in the growth delay experiment. At 25 mg kg$^{-1}$, the combined effect with radiation was marked and possibly more than additive, but the effect of emitefur alone was not as marked as the result of the five-fraction growth delay experiment. Such discrepancies between the results of the two assays may be partly due to the tumour types and numbers of treatments, but they may also be due to the assay. As shown in Figures 2 and 3, the effect of emitefur was manifested immediately after administration, which was in marked contrast with the delayed effect of radiation. In this assay, the tumours were excised about 44 h after the first irradiation, and it is possible that the effect of the first (and possibly second) dose of emitefur was not reflected in the result. We used this particular assay because EMT6 tumours are not suitable for the growth delay assay because of their immunogenicity; however, growth delay assay with non-immunogenic tumours may better demonstrate the overall effect of anti-cancer agents such as emitefur.

This series of experiments demonstrated that emitefur is effective at clinically relevant dose levels and that a threshold dose for its efficacy exists between 12.5 and 25 mg kg$^{-1}$. Because of the great difference in effect between these two doses, pharmacokinetic monitoring is recommended in clinical studies. As the AUC for 5-FU in humans shortly after starting emitefur administration may be similar to that obtained with 12.5 mg kg$^{-1}$ emitefur in mice, the clinical efficacy may not be expected to manifest at such an early period. However, reasonable anti-tumour and radiation-enhancing effects may be expected after several days of emitefur administration, when the 5-FU AUC has reached higher levels.

This study also disclosed that the effect of emitefur becomes greater with increasing number of emitefur treatments. In the next clinical studies, 200 mg of emitefur will be given twice daily for 2 weeks, after which the drug will be discontinued for 2 weeks to allow recovery from myelosuppression, and then the cycle will be repeated. From the results of our study, we expect that such administration for 14 consecutive days will be more effective than fewer treatments during a cycle.

In summary, emitefur seems to be quite an effective anticancer and radiation-potentiating agent, even at doses that seem to be clinically relevant. The interaction between emitefur and radiation seems to be at least additive. Further clinical evaluation is warranted.

**Acknowledgements**
The authors wish to thank Dr Hiroshi Kiyokawa and Mr Takeshi Imaoka for technical assistance.

### References

BUCHHOLZ DJ, LEPEK KJ, RICH TA AND MURRAY D. (1995). 5-Fluorouracil–radiation interactions in human colon adenocarcinoma cells. *Int. J. Radiat. Oncol. Biol. Phys.*, **32**, 1053–1058.

FRAILE RJ, BAKER LH, BUROKER TR, HORWITZ J AND VAITKEVICIUS VK. (1980). Pharmacokinetics of 5-fluorouracil administered orally, by rapid intravenous and by slow infusion. *Cancer Res.*, **40**, 2223–2228.

FUJII S, FUKUSHIMA M, SHIMAMOTO Y, OHSHIMO H, IMAOKA T AND SHIRASAKA T. (1989). Antitumor activity of BOF-A2, a new 5-fluorouracil derivative. *Jpn J. Cancer Res.*, **80**, 173–181.

HARADA M, NISHITANI H, KOGA K, MIURA I AND KIMURA A. (1993). Comparative studies on the metabolism of new fluorinated pyrimidine drugs in the liver by *in vivo* magnetic resonance spectroscopic observation. *Jpn J. Cancer Res.*, **84**, 197–202.

HILLCOAT BL, McCULLOCH PB, FIGUEREDO AT, EHSAN MH AND ROSENFELD JM. (1978). Clinical response and plasma levels of 5-fluorouracil in patients with colonic cancer treated by drug infusion. *Br. J. Cancer*, **38**, 719–724.

HIROHASHI M, KIDO M, YAMAMOTO Y, KOJIMA Y, JITSUKAWA K AND FUJII S. (1993). Synthesis of 5-fluorouracil derivatives containing an inhibitor of 5-fluorouracil degradation. *Chem. Pharm. Bull.*, **41**, 1498–1506.

LAWRENCE TS AND MAYBAUM J. (1993). Fluoropyrimidines as radiation sensitizers. *Semin. Radiat. Oncol.*, **3**, 20–28.

LOKICH JJ, AHLGREN JD, GULLO JJ, PHILIPS JA AND FRYER JG. (1989). A prospective randomized comparison of continuous infusion fluorouracil with a conventional bolus schedule in metastatic colorectal carcinoma: a Mid-Atlantic Oncology Program study. *J. Clin. Oncol.*, **7**, 425–432.

MIYAUCHI S, IMAOKA T, UTSUNOMIYA T, HAYASHI K, KUBO M, KAWAGUCHI T AND MATSUI Y. (1994). Oral administration of BOF-A2 to rats with lung transplanted tumors results in increased 5-fluorouracil levels. *Jpn J. Cancer Res.*, **85**, 665–668.

MIYAUCHI S, IMAOKA T, OKADA T, MOTOYAMA M, KAWAGUCHI T, AKIYAMA H AND ODOMI M. (1996). Leukopenia-inducing effect of a combination of a new 5-fluorouracil (5-FU)-derived drug, BOF-A2 (emitefur), with other 5-FU-derived drugs or BV-araU (Sorivudine) in rats. *Jpn J. Pharmacol.*, **70**, 139–148.

MOERTEL CG, FRYTAK S, HAHN RG, O'CONNELL MJ, REITEMEIER RJ, RUBIN J, SCHUTT AJ, WEILAND LH, CHILDS DS, HOLBROOK MA, LAVIN PT, LIVSTONE E, SPIRO H, KNOWLTON A, KALSER M, BARKIN J, LESSNER H, MANN-KAPLAN R, RAMMING K, DOUGLAS HO JR, THOMAS P, NAVE H, BATEMAN J, LOKICH J, BROOKS J, CHAFFEY J, CORSON JM, ZAMCHECK N AND NOVAK JW. (1981). Therapy of locally unresectable pancreatic carcinoma: a randomized comparison of high dose (6000 rads) radiation alone, moderate dose radiation (4000 rads + 5-fluorouracil), and high dose radiation + 5-fluorouracil. *Cancer*, **48**, 1705–1710.

MURATA R, SHIBAMOTO Y, MIYAUCHI S, HIROHASHI M, TAKAGI T, SASAI K, OYA N AND HIRAOKA M. (1996). The combined antitumour effect of a new 5-fluorouracil derivative, BOF-A2, and radiation in mouse SCCVII tumour. *Br. J. Cancer*, **74** suppl. XXVII, S114–S116.

NAKAI Y, FURUSE K, OHTA M, YAMAGUCHI Y, FUJII M, ASAKAWA M, FUKUOKA M, YOSHIDA K AND NIITANI H. (1994). Efficacy of a new 5-fluorouracil derivative, BOF-A2, in advanced non-small cell lung cancer. A multi-center phase II study. *Acta Oncol.*, **33**, 523–526.

OKAYASU T, SUGIYAMA K AND MIYAUCHI S. (1994). Inhibition of catabolic pathway of 5-fluorouracil by 3-cyano-2,6-dihydroxypryridine in human lung cancer tissues. *Jpn J. Cancer Res.*, **85**, 101–105.

SAKATA Y, YASUTAKE K, NISHITANI H, KAWAGUCHI T, YOSHIDA Y AND SHIMOYAMA T. (1995). Clinical pharmacokinetics and anti-tumor effect comparison of BOF-A2, a new 5-fluorouracil derivative, with continuous venous infusion of 5-fluorouracil in patients with advanced cancer. *Can. J. Infect. Dis.*, **6** (suppl. C), 438C.

SANCHÍZ F, MILLÁ A, TORNER J, BONET F, ARTOLA N, CARREÑO L, MOYA LM, REIRA D, RIPOL S AND CIRERA L. (1990). Single fraction per day versus two fractions per day versus radiochemotherapy in the treatment of head and neck cancer. *Int. J. Radiat. Oncol. Biol. Phys.*, **19**, 1347–1350.

SEIFERT P, BAKER LH, REED ML AND VAITKEVICIUS VK. (1975). Comparison of continuously infused 5-fluorouracil with bolus injection in treatment of patients with colorectal adenocarcinoma. *Cancer*, **36**, 123–128.

SHIBAMOTO Y, KOMURO C, TAKAHASHI M, ONO K AND ABE M. (1985). The effect of $N^6$-butyrylcordycepin on potentially lethal damage repair *in vivo*. *J. Radiat. Res.*, **26**, 404–410.

SHIBAMOTO Y, YUKAWA Y, TSUTSUI K, TAKAHASHI M AND ABE M. (1986). Variation in the hypoxic fraction among mouse tumors of different types, sizes, and sites. *Jpn J. Cancer Res.*, **77**, 908–915.

SHIBAMOTO Y, SASAI K AND ABE M. (1987). The radiation response of SCCVII tumor cells in C3H/He mice varies with the irradiation conditions. *Radiat. Res.*, **109**, 352–354.

SHIRASAKA T, FUJITA F, FUJITA M, FUKUSHIMA M, TAGUCHI T AND FUJII S. (1990). Antitumor activity and metabolism of BOF-A2, a new 5-fluorouracil derivative, with human cancers xenografted in nude mice (in Japanese). *Jpn J. Cancer Chemother.*, **17**, 1871–1876.

TATSUMI K, YAMAUCHI T, KIYONO K, KISHI K, YANAGIHARA Y, IMAOKA T, KAWAGUCHI T AND KUBO M. (1993). 3-Cyano-2,6-dihydroxypyridine (CNDP), a new potent inhibitor of dihydrouracil dehydrogenase. *J. Biochem.*, **114**, 912–918.

WEINBERG MJ AND RAUTH AM. (1987). 5-Fluorouracil infusions and fractionated doses of radiation: studies with a murine squamous cell carcinoma. *Int. J. Radiat. Oncol. Biol. Phys.*, **13**, 1691–1699.